# Functional Differences of Grapevine Circular RNA *Vv-circPTCD1* in *Arabidopsis* and Grapevine Callus under Abiotic Stress

**DOI:** 10.3390/plants12122332

**Published:** 2023-06-15

**Authors:** Yi Ren, Junpeng Li, Jingjing Liu, Zhen Zhang, Yue Song, Dongying Fan, Minying Liu, Lipeng Zhang, Yuanyuan Xu, Dinghan Guo, Juan He, Shiren Song, Zhen Gao, Chao Ma

**Affiliations:** 1 Shanghai Collaborative Innovation Center of Agri-Seeds, School of Agriculture and Biology, Shanghai Jiao Tong University, Shanghai 200240, China; renyi426@sjtu.edu.cn (Y.R.); dirklee41@sjtu.edu.cn (J.L.); zhangzhen_1217@sjtu.edu.cn (Z.Z.); song_yue@sjtu.edu.cn (Y.S.); fandongying@sjtu.edu.cn (D.F.); luciana_liu@sjtu.edu.cn (M.L.); xyy19971004@sjtu.edu.cn (Y.X.); guodinghan1997@sjtu.edu.cn (D.G.); juan.he@sjtu.edu.cn (J.H.); sr.song@sjtu.edu.cn (S.S.); 2 Department of Horticulture, College of Agriculture, Shihezi University, Shihezi 832003, China; jingjing@stu.shzu.edu.cn (J.L.); zlp@stu.shzu.edu.cn (L.Z.); 3 State Key Laboratory of Crop Biology, College of Horticulture Science and Engineering, Shandong Agricultural University, Taian 271018, China; gaoz89@sdau.edu.cn

**Keywords:** grapevine, abiotic stress, noncoding RNA, circRNA, pentatricopeptide repeat proteins, back-splicing

## Abstract

Circular RNAs (circRNAs) serve as covalently closed single-stranded RNAs and have been proposed to influence plant development and stress resistance. Grapevine is one of the most economically valuable fruit crops cultivated worldwide and is threatened by various abiotic stresses. Herein, we reported that a circRNA (*Vv-circPTCD1*) processed from the second exon of the pentatricopeptide repeat family gene *PTCD1* was preferentially expressed in leaves and responded to salt and drought but not heat stress in grapevine. Additionally, the second exon sequence of *PTCD1* was highly conserved, but the biogenesis of *Vv-circPTCD1* is species-dependent in plants. It was further found that the overexpressed *Vv-circPTCD1* can slightly decrease the abundance of the cognate host gene, and the neighboring genes are barely affected in the grapevine callus. Furthermore, we also successfully overexpressed the *Vv-circPTCD1* and found that the *Vv-circPTCD1* deteriorated the growth during heat, salt, and drought stresses in *Arabidopsis*. However, the biological effects on grapevine callus were not always consistent with those of *Arabidopsis*. Interestingly, we found that the transgenic plants of linear counterpart sequence also conferred the same phenotypes as those of circRNA during the three stress conditions, no matter what species it is. Those results imply that although the sequences are conserved, the biogenesis and functions of *Vv-circPTCD1* are species-dependent. Our results indicate that the plant circRNA function investigation should be conducted in homologous species, which supports a valuable reference for further plant circRNA studies.

## 1. Introduction

Noncoding RNAs have increasingly crucial functions for growth, development, and stress response in plants [1,2]. Circular RNAs (circRNAs), which have not been found to have the coding ability in plants yet, are single-stranded noncoding RNAs that are processed by backing-splicing in which a downstream 5׳ splice site is linked by a 3′,5′- phosphodiester bond to an upstream 3′ splice site in a reverse order crossing one exon or exons [3]. Owing to the development of high-throughput sequencing technology and associated analytical tools, thousands of circRNAs have been identified in various plants and deposited in an associated database [4,5]. At present, it has been reported that a total of 171,118 circRNAs from 21 plant species have been collected in PlantcircBase [6]. The biological functions of circRNA have been well investigated in mammals. Animal circRNAs have generally been suggested to function as miRNA sponges for decaying endogenous miRNA [7], as templates for translation [8,9], as mediators for regulating assembly and activation of the AMPK complex under stress [10], and as enhancers for cis-regulation of their host genes [11]. In plants, previous studies have focused on the identification and annotation of putative circRNAs as well as function investigations. Until now, many studies have provided direct evidence that circRNAs have important functions in plants through overexpression and gene editing technologies. Overexpression of circRNA derived from *PSY1* (*Phytoene Synthase 1*) and *PDS* (*Phytoene Desaturase*) in tomato, respectively, causes color variation by reducing the accumulation of lycopene and *β*-carotene; however, the regulatory network is not uncovered [12]. In *Arabidopsis*, overexpression of *circSEP3* results in floral organ abnormalities. *CircSEP3* from exon six of *SEPALLATA3* can bind strongly to its homological genomic region of the host gene, forming an R-loop structure, whereas the linear RNA equivalent bound significantly weakly to DNA. R-loop formation leads to transcriptional pausing, which regulates exon-skipped alternative splicing of its host gene [13]. CRISPR-Cas9 strategy has been applied to remove *Os06circ02797* processing by editing the flanking sequence in rice, resulting in a rapid growth phenotype and higher chlorophyll A/B content, in which *Os06circ02797* was supposed to bind and sequester *OsMIR408*. A circRNA–miRNA-mRNA regulatory network was genetically proved in plants, but the biochemical evidence is limited [14]. In *Populus tomentosa*, overexpression of *Circ_0003418* derived from a RING-type E3 ligase gene *XBAT32* deteriorates the callus thermotolerance. Here, the *Circ_0003418* positively regulated its host gene and specifically increased the expression of the alternative transcript variant, which lacks the E3 ubiquitin ligase domain [15]. In *Arabidopsis*, an antisense *ag-circRBCS* significantly inhibited the expression of RBCS, which is a RuBisCO small subunit [16]. Lately, mitochondrion-derived circRNAs (mcircRNAs) have been characterized in plants such as maize, *Arabidopsis*, rice, tomato, cucumber, and grape. Interestingly, based on translational profile data, many mcircRNAs bound to ribosomes were detected in maize and *Arabidopsis,* and proteomics data found 358 mcircRNA-derived polypeptides. Those results implied the circRNA potentially has coding ability, whereas the translational initiation mechanism is unclear [17]. Although some studies have been performed, functional investigation of circRNAs in plants is still very limited. Therefore, additional research needs to be conducted.

Grapevine (*Vitis* spp.) is the most economically important fruit crop cultivated worldwide for the purpose of producing table grapes, dried fruits, and raw materials for juice and winemaking [18]. The growth, development, and flavor of berries are strongly influenced by various abiotic stresses, such as high temperature, water deficit, and salt during viticulture [19,20,21]. Previous findings suggested that high temperature influences grapevine photosynthesis, fluorescence, and veraison [22,23,24]. In grapevine, the serine/arginine-rich splicing factors (SR) are generally phosphorylated at high temperatures, which results in the disturbance of alternative splicing of pre-mRNA [25,26]. The mechanisms involved in grapevine tolerance to high temperatures are poorly understood, although the heat shock factors (HSFs)-mediated regulatory networks have been well deciphered in other plants [27,28,29]. Drought is another important environmental factor limiting grapevine growth and productivity, and studies have shown that the photosynthesis of vegetative organs, berry size and yield, and accumulation of secondary metabolites are greatly affected by the water deficit [30]. Abscisic acid (ABA) is induced and plays a key role in response to water deficits by regulating stomatal movements, and the signaling pathway has been well-studied in plants [31]. Furthermore, the long noncoding RNAs (LncRNA) and microRNAs (miRNA) also serve as novel regulators of the plant response to drought stress in *Arabidopsis* [32], *Brassica napus* L. [33], and grapevine [34]. Salt stress is another factor that is mainly due to the hyperosmotic conditions of soil solution with an increase of Na^+^ and Cl^−^, causing the deficiency of absorption in water and nutrients in plants [35]. In grapevine, previous studies found that many stress-induced genes, such as *VvWRKY2*, *VvNAC17*, and *VvASMT1*, perform functions for enhancing salt and osmotic stress tolerance in ectopic overexpression [21,36,37]. Although the above findings greatly uncover the molecular mechanisms responding to abiotic stress in grapevines, the function of circRNAs also needs to be further explored.

In grapevine, according to our previous study, a total of 8,354 circRNAs have been identified by whole transcriptome analysis, and a *Vv-circATS1* spliced from *glycerol-3-P acyltransferase* was proven to improve cold tolerance in *Arabidopsis* [38]. In the present study, a circRNA derived from the second exon of the *pentatricopeptide repeat domain-containing protein 1* (*PTCD1*, VIT_211s0016g03830) was verified based on the grapevine circRNA dataset, named *Vv-circPTCD1*. Pentatricopeptide repeat (PPR) protein is a large gene family in plants that are characterized by 2–15 tandem repeats of 30–40 amino acid length motifs. Some PPR proteins have been supposed to play roles in organellar RNA metabolism, organ development, as well as stress response [39]. Overexpression of *Vv-circPTCD1* deteriorated the tolerance to heat, salt, and PEG-mediated osmotic stresses in *Arabidopsis*. While similarly, the linear fragment identical to the circRNA sequence also did. However, the *Vv-circPTCD1* overexpressed callus mass of ‘Thompson Seedless’ failed to display the phenotypes consistent with *Arabidopsis*. Those results implied that the functions of *Vv-circPTCD1* were potentially sequence-dependent and not conserved among plants.

## 2. Results

### 2.1. Identification and Verification of Vv-circPTCD1

**Figure 1:**
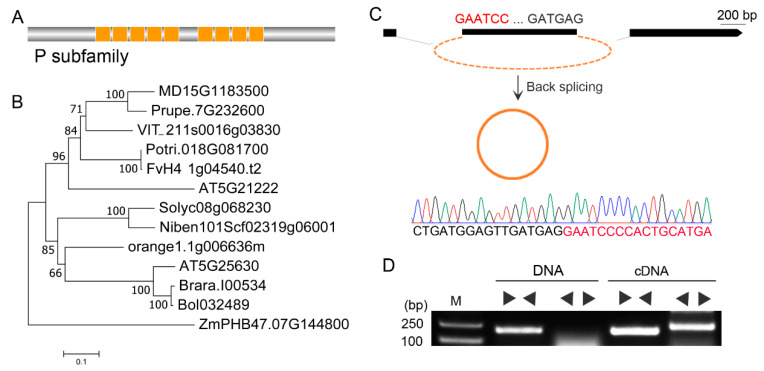
Identification of *Vv-circPTCD1* in grapevine. (**A**) The graphical representation of the PTCD1 protein; (**B**) The phylogenetic analysis of *PTCD1* (VIT_211s0016g038030.1) with orthologs in other species. (**C**) Verification of *Vv-circPTCD1* processed in the second exon. (**D**) PCR amplification of divergent and convergent primers based on DNA and cDNA in grapevine. “►◄” indicated the convergent primer pairs, and “◄►” indicated the divergent primer pairs.

Based on the previously published circRNA dataset of ‘Muscat Hamburg’ [38], a circRNA_4363, back-spliced from the second exon of the pentatricopeptide repeat (PPR) proteins family gene *PTCD1*, was identified and renamed *Vv-circPTCD1* in this research. *PTCD1* encodes a protein with 636 amino acid residues in length and contains the nine tandem PPR domains (Figure 1A). In the *Arabidopsis* genome, meanwhile, two orthologs (AT5G21222 and AT5G25630) were characterized, and phylogenetic analysis showed that AT5G21222 and *PTCD1* (VIT_211s0016g038030.1) were clustered into one clade with 96% confidence (1000 replicates) (Figure 1B). The *Vv-circPTCD1* was further verified by cloning and sequencing, and the back-splicing site was AG/GA (Figure 1C). Divergent and convergent primers were designed to amplify the target fragment from DNA and leaf cDNA, and the results showed that the fragment with the back-splicing site was only detected in cDNA templates (Figure 1D).

### 2.2. Nonconservative Back-Splicing of Vv-circPTCD1 in Plants

To investigate whether *Vv-circPTCD1* was conserved in plants, we analyzed the physical gene structure of *PTCD1* in plants, including monocots and dicots. The result showed that similar physical gene structures of *PTCD1* orthologs were observed (Figure S1), and a CDS (coding sequence) back-spliced into *Vv-circPTCD1* was found in grapevines, which was highly conserved by multiple sequence alignment among plants (Figure S2). It was reasonably hypothesized that this circRNA orthologous with *Vv-circPTCD1* might also be conserved in plants. Therefore, the divergent primer pair was tentatively designed to clone the orthologs in *Arabidopsis* and *N. benthamiana*, although non-specific amplification was found in *N. benthamiana*, while the *Vv-circPTCD1* orthologs failed to be cloned (Figure S3). Those results suggested that the back-splicing of *Vv-circPTCD1* was potentially species-dependent.

### 2.3. The Expression Analyses of PTCD1 and Vv-circPTCD1 under Abiotic Stress

**Figure 2:**
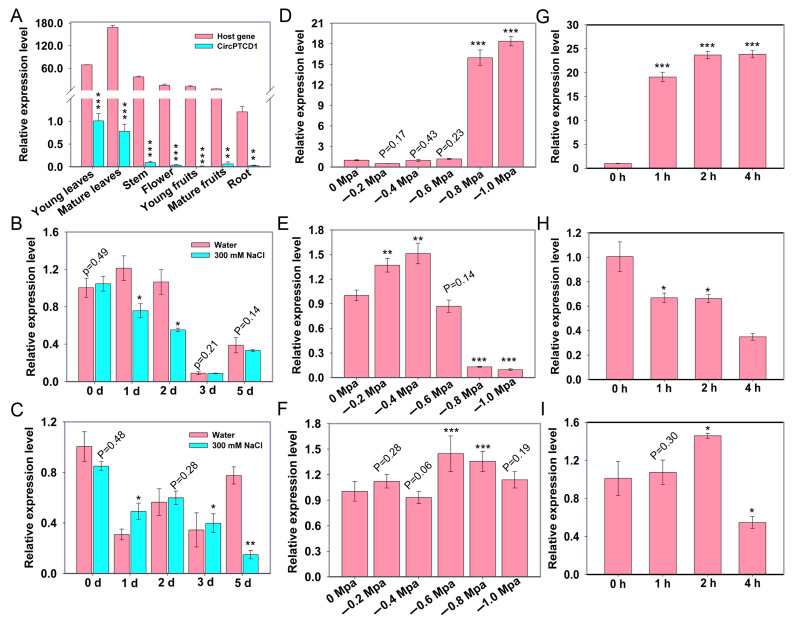
Expression patterns of *PTCD1* and *Vv-circPTCD1* under stress. (**A**) The tissue-specificity analyses of *PTCD1* and *Vv-circPTCD1*; (**D**,**G**) The expression pattern of marker genes *VvSnRK2I* and *VvHsfA7* under drought and heat treatment; (**B**,**E**,**H**) The expression pattern of *PTCD1* during salt, drought, and heat stress; (**C**,**F**,**I**) The expression pattern of *Vv-circPTCD1* during salt, drought, and heat stress. Differences between mean values of groups are compared using a *t*-test (“*”: *p* < 0.05; “**”: *p* < 0.01; “***”: *p* < 0.001). The data are presented as the mean ± SE.

To investigate the potential functions in grapevine, we analyzed the tissue specificity and the expression patterns of *Vv-circPTCD1* and its corresponding host gene under salt, heat, and drought stress conditions, respectively. The *Vv-circPTCD1* was mainly expressed in young and mature leaves while being scarcely expressed in roots, consistent with that of the host gene (Figure 2A). However, the accumulation level of the host gene was excessively higher than that of *Vv-circPTCD1* (more than sixty times) (Figure 2A). During salt stress, in addition to the host gene being mildly downregulated during initial treatment, the *Vv-circPTCD1* was significantly downregulated on the fifth day after treatment (Figure 2B,C). During drought treatment, *VvSnRK2I* (VIT_207s0197g00080), a marker gene homologous with *AtSRK2I*/*AtSnRK2.3* induced by ABA in *Arabidopsis* [40], was significantly induced when the soil water potential reduced to −0.8 and −1.0 MPa (Figure 2D). The expression level of *PTCD1* was mildly upregulated when the water potential was −0.4 MPa, while the expression level of *PTCD1* was significantly reduced when the water potential was reduced to −0.8 and −1.0 MPa (Figure 2E). However, the expression pattern of *Vv-circPTCD1* barely changed during drought treatment (Figure 2F). We further investigated the expression pattern during heat stress, and the results showed that the heat shock factor genes *VvHsfA7*, as a marker of heat stress [22], were remarkably upregulated (Figure 2G), and the expression level of *PTCD1* was downregulated with the prolongation of treatment (Figure 2H). Although the expression level of *Vv-circPTCD1* was downregulated after 4 h of treatment, the expression level was also slightly upregulated after 2 h of treatment (Figure 2I). Those results indicated that the *Vv-circPTCD1* expression always fell behind that of *PTCD1* and potentially played a role during abiotic stresses in grapevines.

### 2.4. Overexpression of Vv-circPTCD1 in Grapevine Callus and Arabidopsis

**Figure 3:**
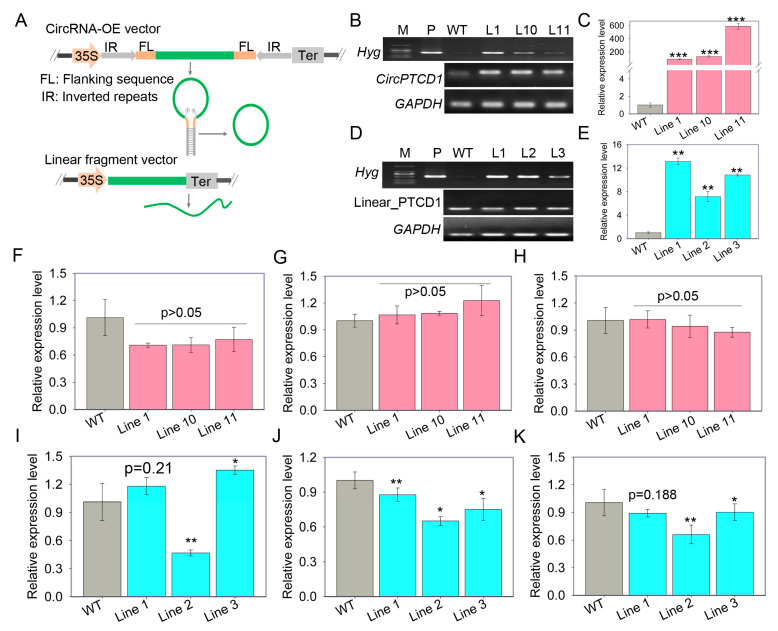
The OE of *Vv-circPTCD1* and corresponding linear fragment (Linear_PTCD1) in the callus of ‘Thompson Seedless’ (**A**) The schematic of vector construction for circRNA and linear fragment OE, the green line represents the sequence back-spliced into circRNA, and the CaMV35S (35S) promoter was used to initiate transcription, Ter refers to transcription termination; (**B**,**C**) The PCR and RT-qPCR confirmation of *Vv-circPTCD1* OE in grapevine callus, three positive callus masses were identified, *Hyg* encode the hygromycin phosphotransferase responsible for resistance selection; (**D**,**E**) the PCR and RT-qPCR confirmation of Linear_PTCD1 OE in grapevine callus; (**F**–**H**) The expression level of the host gene, upstream gene, and downstream gene in *Vv-circPTCD1* OE lines; (**I**–**K**) The expression level of the host gene, upstream gene, and downstream gene in Linear_PTCD1 OE callus mass. The red bar chart means the *Vv-circPTCD1* OE callus, the turquoise bar chart means the Linear_PTCD1 OE callus. Differences between mean values of groups are compared using a *t*-test (“*”: *p* < 0.05; “**”: *p* < 0.01; “***”: *p* < 0.001). The data are presented as the mean ± SE.

To decipher the functions of *Vv-circPTCD1* in plants, the vector for *Vv-circPTCD1* overexpression (OE) was generated. Meanwhile, a corresponding linear fragment (Linear_PTCD1) identical to *Vv-circPTCD1* was also constructed as a control (Figure 3A). The overexpressed callus mass was verified by RT-PCR and RT-qPCR, respectively (Figure 3B–E). The expression level of the host gene and flanking genes were further investigated in all OE callus masses. Among *Vv-circPTCD1* lines, the expression level of the host gene was slightly downregulated (Figure 3F), while the upstream gene (VIT_211s0016g03850) and downstream gene (VIT_211s0016g03820) were not significantly affected (Figure 3G,H). However, among Linear_PTCD1 OE lines, the expression level of the host gene was discrepant among different lines, which is downregulated in line 2 (Figure 3I). The upstream gene and downstream gene were also slightly downregulated (Figure 3J,K). In order to uncover whether the function of *Vv-circPTCD1* is conserved among plants, ectopic transformation was also performed in *Arabidopsis* (Figure S4).

### 2.5. Phenotype of OE Lines under Heat Stress

**Figure 4:**
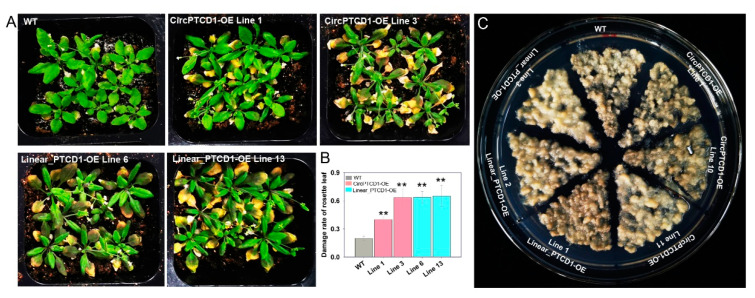
The phenotype of OE lines in *Arabidopsis* and the callus of grapevine under heat stress. (**A**) Deteriorated phenotype of transgenic *Arabidopsis*; (**B**) Damage rate of rosette leaves in overexpressed *Arabidopsis*; (**C**) The phenotype of overexpressed callus under heat stress. Differences between the mean values of groups were compared using a *t*-test (“**”: *p* <0.01).

To investigate whether *Vv-circPTCD1* plays a role in *Arabidopsis*, the four-week-old seedlings were treated for 12 h at 45 °C followed by exposure to 22 °C for 7 days to recover. Results showed that compared with WT, the dead rosette leaves were significantly increased in *Vv-circPTCD1* OE lines (CircPTCD1-OE), and the reproductive growth was unaffected (Figure 4A). We also counted the death rate of rosette leaves in *Arabidopsis* and found that the damage rate of rosette leaves was significantly increased in transgenic lines (Figure 4B). It was a wonder that the rosette leaves were also remarkably damaged in linear fragment OE lines (Linear_PTCD1-OE), which keep an identical sequence with *Vv-circPTCD1* (Figure 4A,B). We also observed the phenotype of transgenic callus responding to heat stress, in which the callus was continuously incubated for 15 d at 40 °C. We found that all calluses became brown; however, phenotypic differences were not significant, no matter which OE callus mass (Figure 4C and Figure S5). Those results imply that the function of *Vv-circPTCD1* is potentially not conserved in plants.

### 2.6. Phenotype of OE Lines under Salt Stress

**Figure 5:**
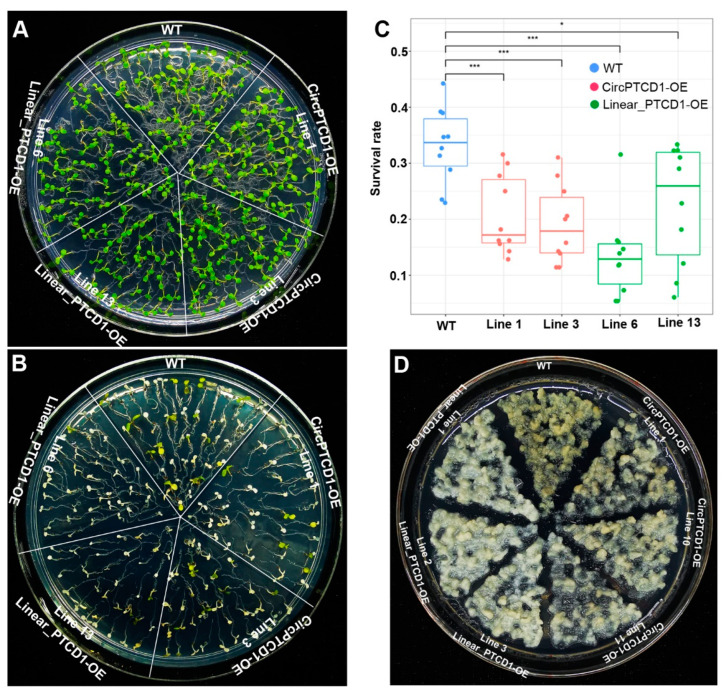
The phenotype of OE lines in *Arabidopsis* and the callus of grapevine under salt stress. (**A**) The phenotype of OE seedlings and WT on 1/2 MS medium; (**B**,**C**) The phenotype and survival rate of OE seedlings and WT on 1/2 MS medium containing 300 mM NaCl; (**D**) The phenotype of OE and WT callus mass incubated on medium containing 300 mM NaCl. Differences between mean values of groups are compared using a *t*-test (“*”: *p* < 0.05; “***”: *p* < 0.001).

To further survey the potential function of *Vv-circPTCD1* in plants, four-day-old seedlings of *Arabidopsis* were transplanted into normal and NaCl-containing media to observe the variation. Results suggested that different OE lines and WT seedlings were normally and identically grown in NaCl-free medium (Figure 5A), while the survival rate of transgenic lines (CircPTCD1-OE) was lower on the NaCl-containing medium after five days compared to WT, although most *Arabidopsis* seedlings died after salt stress (Figure 5B,C). The OE lines of the linear fragment (Linear_PTCD1-OE) were also severely influenced and had a lower survival rate on NaCl-containing media (Figure 5B,C). In grapevine callus, the OE callus mass transformed with linear fragment adversely displayed an albino phenotype, while the OE callus mass transformed with *Vv-circPTCD1* also did but much slighter compared to the WT callus mass (Figure 5D). Those results also showed the *Vv-circPTCD1* potentially served as a negative factor for salt stress, and the linear fragment identical with the *Vv-circPTCD1* also plays the same roles in *Arabidopsis* and grapevine callus.

### 2.7. Phenotype of OE Lines under Drought Stress

**Figure 6:**
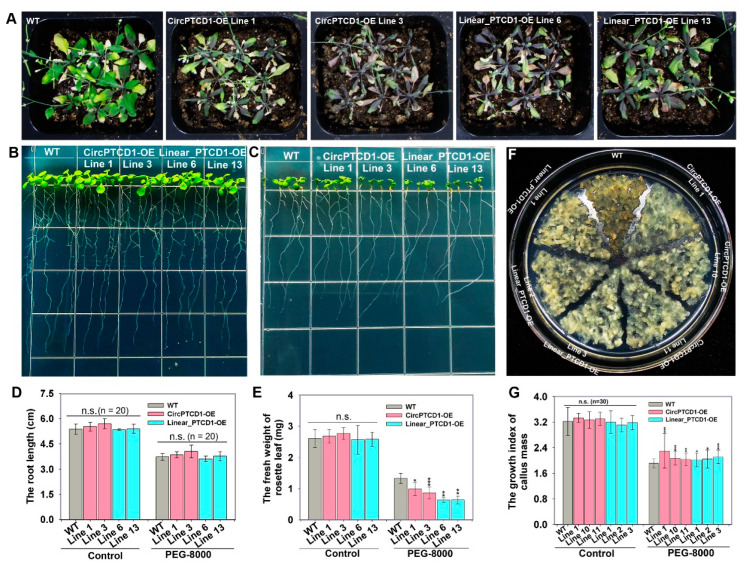
The phenotype of OE lines in *Arabidopsis* and callus of grapevine under drought stress. (**A**) The phenotype of a month-old *Arabidopsis* during drought stress; (**B**) The growth of seedlings incubated on PEG-free medium; (**C**) The growth of seedlings incubated on medium containing PEG-8000; (**D**,**E**) The root length and fresh weight of rosette leaves of seedlings during PEG-8000 treatment; (**F**) The phenotype of grapevine callus mass incubated on medium containing PEG-8000; (**G**) The growth index of callus mass incubated on medium containing PEG-8000. Differences between mean values of groups are compared using a *t*-test (“*”: *p* < 0.05; “**”: *p* < 0.01; “***”: *p* < 0.001; n.s.: *p* > 0.05). The data are presented as the mean ± SE.

To comprehensively explore the function of *Vv-circPTCD1* in *Arabidopsis*, the four-week-old seedlings were stopped from watering. Results showed that the transgenic lines of *Vv-circPTCD1* (CircPTCD1-OE) were much more susceptible and withered, and the leaves turned purple due to the accumulation of anthocyanins during drought stress (Figure 6A). The OE lines of the linear fragment (Linear_PTCD1-OE) also turned purple and withered during drought stress compared to the WT (Figure 6A). The four-day-old seedlings were further transplanted into normal and PEG-containing medium, and it was found that different transgenic lines (CircPTCD1-OE and Linear_PTCD1-OE) and WT seedlings were normally and identically growing in normal media (Figure 6B), while the growth of rosette leaves was obviously inhibited in *Vv-circPTCD1* and linear fragment OE lines (CircPTCD1-OE and Linear_PTCD1-OE), the growth of roots was barely inhibited (Figure 6C–E). Those results suggested that the *Vv-circPTCD*1 and corresponding linear fragment also play negative roles during drought stress in *Arabidopsis*. In grapevine callus, on the contrary, the OE callus mass of the *Vv-circPTCD1* and linear fragment transgenic callus mass (CircPTCD1-OE and Linear_PTCD1-OE) significantly displayed resistance to osmotic stress mediated by PEG-8000, while the WT had become brown after 30 days of incubation (Figure 6F). We further counted the growth index of callus mass and found that the growth rate of transgenic callus was significantly higher than that of WT (Figure 6G and Figure S5). Those results implied that the function of *Vv-circPTCD1* is potentially species-dependent.

## 3. Discussion

CircRNAs are widespread in plants. Most circRNAs are generated by back-splicing from the exon or exons of the genic primary mRNA; for example, 94.5% are derived from the coding genes in rice, and the remaining are intergenic circRNAs [41]. In addition, the mitochondrion genome is also proven to generate circRNAs, called mitochondrion-encoded circular RNAs (mcircRNAs) [17]. Here, we identified a circRNA spliced from the second exon of *PTCD1*, a PPR family gene, in which it was spliced based on a canonical GU/AG splicing signal [38] (Figure 1C). In general, hundreds of these superfamily proteins encoded by the PPR family genes located in the nuclear and organelle genomes are found in plants and are characterized by multiple tandem arrays of the PPR domain [42]. Previous findings proved that the PPR proteins, which serve as RNA binding proteins, affect cytoplasmic male sterility, embryogenesis, seed development, and stress [43,44,45,46]. Interestingly, the physical gene structure of *PTCD1* orthologs is similar, and the sequence of the CDS back-spliced into *Vv-circPTCD1* is highly conserved among plants (Figures S1 and S2). However, the verification failure of the corresponding circRNA in *Arabidopsis* and *N. benthamiana* implies that *circPTCD1* is not naturally conserved among plants. No matter what mammals and plants are, a factually small proportion of circRNAs are supposed to be conserved, such as the overlap of 26% of circRNAs in both humans and mice based on the syntenic region of the genome [8]. In plants, only 8.7% of circRNAs are genomically conserved in dicotyledonous plants, even that is less than half in the *Oryza* genus [41]. Taken together, *Vv-circPTCD1* is not evolutionarily, although the host genes are conserved. This result partially supports the idea that circRNAs result largely from splicing errors [47,48].

We also successfully constructed the artificial vector containing a reverse complementary sequence pair to generate the circRNA in *Arabidopsis* and grapevine callus according to the previous strategy, although the natural back-splicing of *Vv-circPTCD1* is not conserved (Figure 3A) [38]. Intronic complementary sequences have been found in the flanking sequence of highly expressed circRNA in eukaryotes. In mammals, the exons flanked by the orientation-opposite *Alu* elements, short interspersed elements in primates, are preferentially circularized [49]. In *Populus tomentosa*, miniature inverted-repeat transposable elements (MITEs) are enriched in circRNA flanking regions and serve as a cis-regulatory factor to significantly regulate exon circularization [15]. Furthermore, previous findings suggested that the back-splicing of circRNA is also executed by the canonical spliceosome, a highly conserved precursor mRNA splicing mechanism in eukaryotes [50]. Additionally, the flanking intron sequence is crucial for the precise splicing and circularization of one exon and exons, which has been proven by heterologous investigations [38,51]. Therefore, the inverted repeats and flanking sequence commonly contribute to the fidelity and expression level of *Vv-circPTCD1* in *Arabidopsis* and grapevine callus (Figure 3B,C and Figure S4). Therefore, the artificial “stem-loop” structure mediated by the reverse complementary sequence greatly contributes to the back-splicing of *Vv-circPTCD1* in heterogeneous expression, in which the “stem-loop” brings the splice sites into close proximity to each other [52].

Additionally, previous investigations uncovered that circRNAs are incorporated into chromatin by an R-loop structure that interacts with the nuclear DNA to regulate chromatin stabilization or alternative splicing [13,53]. The host gene and neighboring genes are potentially influenced by overexpressed or deleted circRNA. In rice, a neighboring gene is significantly upregulated at circRNA mutant lines [14]. In this study, *Vv-circPTCD1* has a slightly negative effect on the host gene and barely any effect on the neighboring genes after overexpression of *Vv-CircPTCD1* (Figure 3F–H). In *P. tomentosa*, overexpression of *Circ_0003418* significantly increased the expression abundance of its parental gene [15]. Therefore, circRNA potentially has the ability to function by disturbing the expression of host or neighboring genes in plants.

The expression pattern of circRNAs is also determined by tissue and environmental factors and even genotype [15,54]. We found that the *Vv-circPTCD1* focused in this research was preferentially expressed in young and mature leaves, which is consistent with the expression pattern of the corresponding host gene, while the expression abundance of *Vv-circPTCD1* was less than 2% that of the host gene (Figure 2A). In addition to some circRNAs in animals (almost 10%) [8], the circRNAs barely hold a higher expression level with respect to the linear counterpart and largely obeyed the expression pattern of host genes in plants, such as grapevine [38]. In this study, the expression level of *PTCD1* is significantly inhibited during drought stress, while the expression of *Vv-circPTCD1* is slightly decreased (Figure 2D–F), indicating it plays a role in grapevine responses to drought stress. In addition, the expression level of *Vv-circPTCD1* is remarkably decreased during salt stress after 5 days, while a decrease in the host gene never occurred (Figure 2B,C). Those results imply that the *Vv-circPTCD1* plays a role during abiotic stress, in which the expression of circRNA is potentially controlled by the specific abiotic stress, at least during the processing or degradation pathway [55].

The hypothesis of “splicing error” is indeed uncontradictory with the biochemical activities and biological functions of circRNAs. According to the previous idea, the biogenesis of circRNAs and the regulation of their abundance are managed by various mechanisms, such as cis- or trans-elements, export, and turnover of circRNAs [3]. In this study, although the expression of *Vv-circPTCD1* is related to salt and drought stress, the mechanism is also unclear. For investigation of the biological function, a problem with their circular conformation and sequence overlap with linear cognate mRNAs and incomplete cyclization is difficult to overcome [50]. The strategy to evade this problem is to transform the linear fragment identically with circRNA as a control. Generally, the linear fragment scarcely functions in plants [13,15,38]. In the present research, the linear counterpart is also transformed as a control (Figure 3D,E and Figure S4). Interestingly, the identical stress-induced phenotype is observed between *Vv-circPTCD1* and its linear counterpart in OE lines (Figure 4, Figure 5 and Figure 6). It is putative that (1) the incompletely cyclized transcript plays roles identical to its linear counterpart; (2) the *Vv-circPTCD1* functions in a sequence-dependent manner. We observed that only the phenotype induced by salt stress is conserved between *Arabidopsis* and grapevine callus (Figure 5), and that is distinctly inconsistent during the treatment of heat and PEG (Figure 3 and Figure 6). Coincidentally, the cognate sequence-generated *Vv-circPTCD1* is greatly conserved among multiple species (Figure S2), while the biogenesis is not (Figure S3). Those results imply that the biological functions of circRNA are potentially species-dependent in plants.

## 4. Materials and Methods

### 4.1. Plant Materials and Treatments

For drought treatment of grapevine, 1-year-old ‘Muscat Hamburg’ cuttings were planted in a mixture of loam and sand (1:1, *v*/*v*) in a flowerpot in a greenhouse. Cuttings with four well-developed leaves were used for drought stress. The well-developed leaves were collected when the soil water potential was 0 MPa, −0.2 MPa, −0.4 MPa, −0.6 MPa, −0.8 MPa, and −1.0 MPa, respectively. For heat stress treatment of grapevine, the cuttings were treated at 45 °C, and the leaves were collected at 0 h, 1 h, 2 h, and 4 h, respectively. For salt stress, the cuttings were planted in a chamber (25 °C) and watered with the 300 mM NaCl solution, and the mature leaves were sampled at 0 d, 1 d, 2 d, 3 d, and 5 d. All samples were quickly frozen in liquid nitrogen and then stored at −80 °C. All treatments were carried out on three biological replicates.

*Arabidopsis* ecotype Col-0 was cultured in a chamber under 16 h light/8 h dark conditions with a light intensity of 600 mmol m^−2^ s^−1^ at a temperature of 22 °C. For heat stress treatment of *Arabidopsis*, six-week-old WT and transgenic plants were exposed to heat stress at 45 °C for 12 h, followed by exposure to 22 °C for 7 days to recover. For drought stress treatment, six-week-old WT and transgenic plants were stopped watering until the substrate was completely dry, and the phenotype was observed. The PEG-8000 treatment (the water potential was −0.7 MPa) was performed according to the Verslues׳s descriptions [56]. The four-day-old seedlings were transferred into the plate containing PEG-8000 and cultured in the chamber for another 5 days. For salt treatment, plates containing 1/2 MS salts supplemented with 300 mM NaCl were prepared, and four-day-old seedlings were incubated. The survival rates were counted after 5 days. Three biological replicates were performed.

### 4.2. Validation of circRNA and RT-qPCR

The total RNA of *Arabidopsis* and grapevine callus was extracted by the TRIzol reagent (Sangon Biotech, China), and the total RNA of grapevine leaves was extracted by the CTAB method. In brief, 200 mg of the sample was powdered and transferred into a RNase-free 2 mL tube. Then, added quickly was 900 μL pre-heated (65 °C) CTAB buffer (2% CTAB, 100 mM Tris-HCl, 20 mM EDTA, 2 M NaCl, 2% PVP-40, pH8.0) and 20 μL *β*-mercaptoethanol. The mixture was mixed robustly and then bathed at 65 °C for 15 min. The lysate was extracted with 900 uL of chloroform: isoamylol (24:1, *v*/*v*) by vortexing for 30 s and centrifuged at 4 °C for 10 min at 12,000 rpm. This was repeated two times with chloroform: isoamylol extraction, 20 μL NaAC (pH5.2) was added, and then 60 μL pre-cooled (−20 °C) absolute alcohol, and bathed 10 min on ice. Then an equal volume chloroform: isoamylol was added before vortexing. The sample was centrifuged at 4 °C for 10 min at 12,000 rpm, then 150 μL 10 M LiCl was added to 450 μL of supernatant and mixed. The RNA was precipitated at 4 °C for 6 to 8 h and harvested by centrifugation at 4 °C for 10 min at 12,000 rpm. Then we carefully removed the supernatant and washed it two times with 70% pre-cooled (−20 °C) alcohol. The pellet was dried and dissolved by 30 μL DEPC-treated ddH_2_O.

The total RNA was transcribed to cDNA using random primers by a FastKing RT Kit (TIANGEN, China) in accordance with the manufacturer’s instructions. To confirm the grapevine circRNAs predicted, a divergent primer was designed by Primer 5 (Table S1). PCR was as follows: 94 °C for 3 min; 40 cycles at 94 °C for 30 s, 56 °C for 15 s, and 72 °C for 20 s; and then 1 cycle at 72 °C for 5 min. For PCR, 2×Taq master mix (Vazyme, China) was used. The PCR products were separated by agarose gel electrophoresis and then purified. Sanger sequencing was further performed to verify the back-spliced junction sites. RT-qPCR (real-time quantitative PCR) analysis was conducted to evaluate the expression levels of circRNAs and linear counterparts using Talent qPCR PreMix (TIANGEN, China) with a qTOWER^3^ Real-Time PCR Detection System (Analytikjena, Germany). Gene expression levels were calculated by the 2^−∆∆Ct^ method [57]. For each RT-qPCR assay, three biological duplicates were conducted.

### 4.3. Vector Construction

All expression vectors were constructed based on the pHB binary plasmids. The construction of the circRNA expression vector was conducted based on a previously published strategy [38]. For the linear fragment expression vector, the linear fragment derived from the same sequence with circRNA was cloned into the pHB vector, which was digested by *Bam*H I and *Xba* I. The constructed plasmids were individually transformed into *Agrobacterium tumefaciens* GV3101 strains and incubated at 28 °C for 2–3 d on LB medium supplemented with 25 mg·L^−1^ rifampicin (Rif) and 50 mg·L^−1^ kanamycin (Kan). A single clone was incubated at 200 rpm and 28 °C for 8–10 h in LB liquid medium containing 25 mg·L^−1^ Rif and 50 mg·L^−1^ Kan. The presence of the corresponding plasmid was verified by PCR. An equal volume of 50% glycerol was added to the positive clone and stored at −80 °C.

### 4.4. Arabidopsis Transformation and Verification

*Arabidopsis* transformation was performed according to the floral dip method [58]. T1 seedlings were selected by spraying Glufosinate ammonium 10% solution (dilute 1000×) (Sangon Biotech, Shanghai, China). The positive transgenic ones were transferred into pots and confirmed by PCR detection using the gene primers. For circRNAs confirmation, the cDNA was used as templates for the PCR method and RT-qPCR reaction, and the splicing site was verified by further sequencing.

### 4.5. Callus Transformation of ‘Thompson Seedless’ and Treatment

The embryogenic callus was induced from floral explants of ‘Thompson Seedless’ according to a previously published protocol [59]. The callus mass was subcultured monthly in MSTP medium (MS base salts, 20 g·L^−1^ sucrose, 1 mg·L^−1^ TDZ, 2.2 mg·L^−1^ picloram, pH5.8) in dark condition at 26 °C. For callus transformation, the *A. tumefaciens*-containing vector was inoculated in 20 mL of LB liquid medium and incubated until OD_600_ reached 1.0. After centrifugation for 5 min at 6000 rpm at 25 °C, the medium was discarded. The pellet was resuspended, and the OD_600_ was adjusted to 0.4 with liquid MS medium (0.1 mM acetosyringone, pH5.8). The bacterial mixture was incubated with the embryogenic callus for 30 min at 40 rpm in a 50 mL conical flask and was then transferred onto filter paper to fully remove and evaporate excess liquid. Then, the callus was collected and transferred to solid MS medium (0.1 mM acetosyringone, 8g·L^−1^ agar, pH5.8) and co-cultivated in dark conditions at 26 °C for 3 days. After 3 days, the callus was transferred to solid MSTP medium containing 50 mg·L^−1^ hygromycin, and 200 mg·L^−1^ Timentin. Petri dishes were subcultured monthly in the dark for the induction of transgenic callus. The expression of circRNA in transgenic callus was confirmed by PCR and RT-qPCR using divergent primer pairs.

For heat stress treatment, the callus mass was subcultured into MSTP medium for one week at 26 °C and then further cultured at 40 °C for 15 days. The callus mass was subcultured into MSTP medium supplemented with 300 mM NaCl for salt stress at 26 °C. For PEG-8000 treatment, the plates were prepared according to the above description, of which the base salt was replaced by MSTP medium supplemented with 15 g·L^−1^ agar on the solid layer, but the liquid layer was the MSTP medium without sucrose and agar. Then the callus mass was subcultured on the PEG-containing plates and cultured at 26 °C for one month. For measuring the growth index of callus mass, the callus was shaped into small pellets (about 3 mm in size), subcultured on the PEG-containing plates, and immediately photographed. After one month of culturing, the plates were also photographed. The area of the pellet was analyzed by Image J software (https://imagej.net/downloads). The growth index of callus mass was the ratio of the area of the pellet after and before culturing. Three biological replicates were performed.

### 4.6. Statistical Analysis and Gene Structure

Data were analyzed statistically using the SPSS 19.0 software. Significant differences were determined using a test; *p* < 0.05 was considered statistically significant. The gene structure was generated by the GSDS 2.0 web tools in Figure S1 [60].

## 5. Conclusions

According to this result, although the cognate counterpart mRNA is greatly conserved among plants, the biogenesis of circRNA generated from them potentially is not. Furthermore, the biological effects of circRNA are largely species-dependent.

## Data Availability

No new datasets were generated or analyzed in this study.

## Supplementary Materials

The following supporting information can be downloaded at: https://www.mdpi.com/article/10.3390/plants12122332/s1, Figure S1: The physical gene structure of *PTCD1 (VIT 211s0016g03830)* orthologs in plants. Figure S2: The multiple sequence alignment of the second exon of PTCD1 orthologs. Figure S3: PCR detection of putative *Vv circPTCD1* ortholog candidates in *Arabidopsis* and *N. benthamiana*. Figure S4: Verification of *Vv circPTCD*1 and Linear_PTCD1 OE lines in Arabidopsis (**A**) The gel electrophoresis of PCR amplification; (**B**) confirmation of back splicing. Figure S5: The phenotype of callus incubated in normal condition. The overexpressed callus mass and WT were incubated at 26 °C on the dark and the phenotypic difference was undetectable.
