# Supplementary material for: Functional Differences of Grapevine Circular RNA Vv-circPTCD1 in Arabidopsis and Grapevine Callus under Abiotic Stress
Source: Plants (Basel). 2023 Jun 15;12(12):2332. doi: 10.3390/plants12122332 (PMC10304202; doi:10.3390/plants12122332)
Supplement: Supplementary file 1 [file plants-12-02332-s001.zip › plants-2381022-supplementary.pdf]

Supplementary files

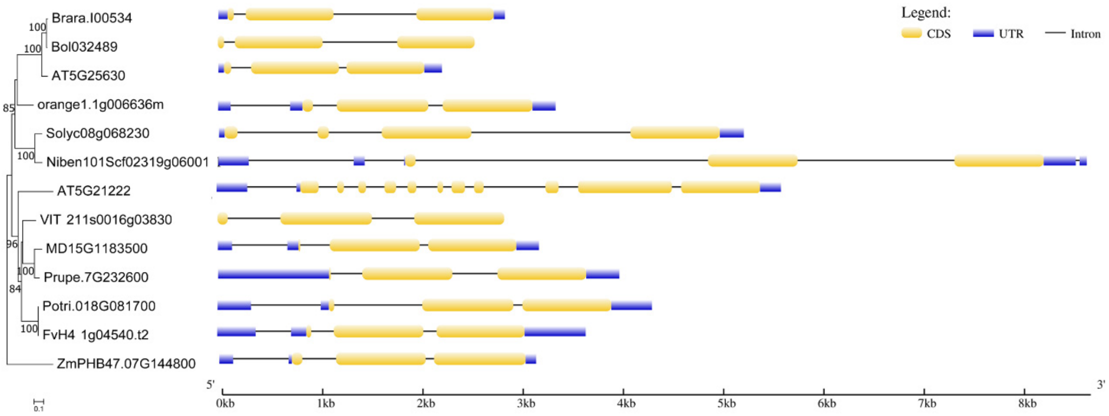

Figure S1. the physical gene structure of *PTCD1* (VIT_211s0016g03830) orthologs in plants. The *PTCD1* orthologs were collected from *Brassica rapa* (Brara.I00534), *Brassica oleracea* (Bol032489), *Arabidopsis thaliana* (AT5G25630 and AT5G21222), *Citrus sinensis* (orange1.1g006636m), *Solanum lycopersicum* (Solyc08g068230), *Nicotiana benthamiana* (Niben101Scf02319g06001), *Malus domestica* (MD15G1183500), *Prunus persica* (Prupe.7G232600), *Populus trichocarpa* (Potri.018G081700), *Fragaria vesca* (FvH4_1g04540.t2), *Zea mays* (ZmPHB47.07G144800). The gene structure was generated by the GSDS 2.0 web tools [60].

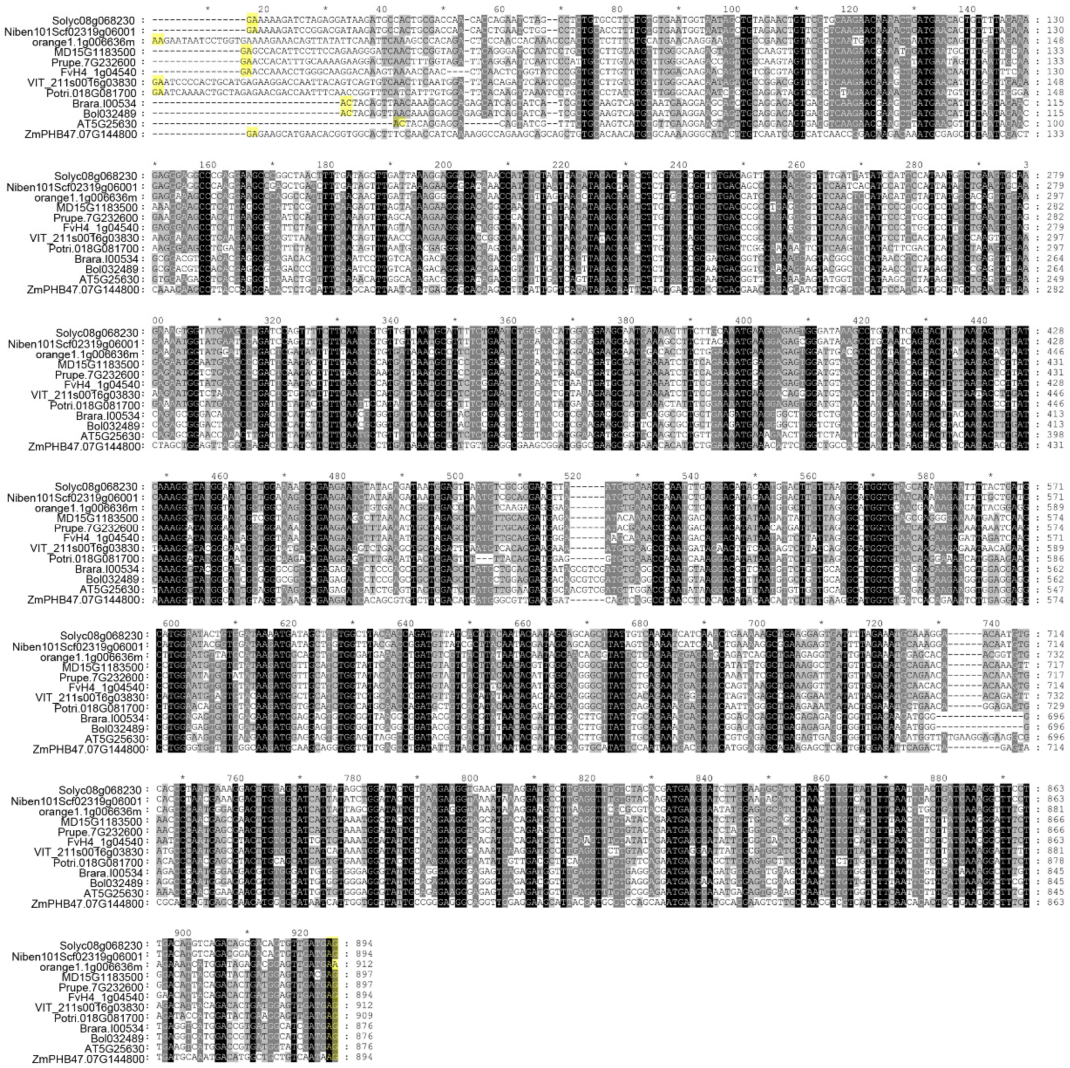

Figure S2. the multiple sequence alignment of the second exon of *PTCD1* orthologs. The accession number of each gene was listed in Figure S1, and the sequence alignment was conducted by ClustalW (https://www.genome.jp/tools-bin/clustalw). The yellow background was the splicing site of exon edge.

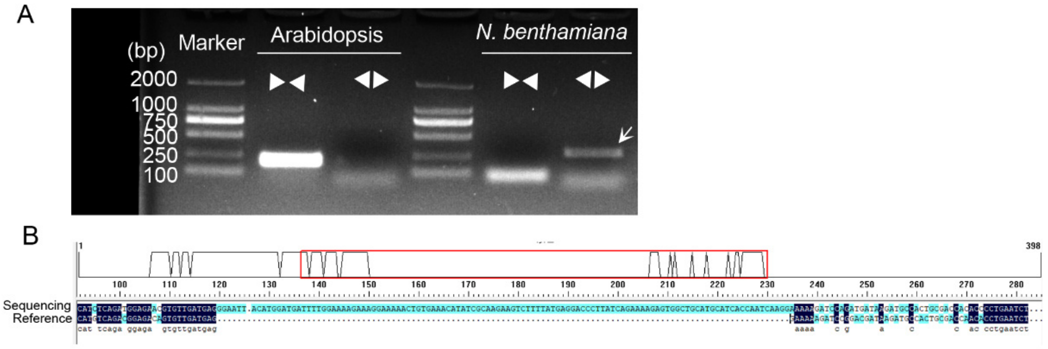

Figure S3. PCR detection of putative *Vv-circPTCD1* ortholog candidates in *Arabidopsis* and *N. benthamiana* (A). The gel electrophoresis imaging of PCR results; (B) Sequence alignment between reference and PCR product derived from *N. benthamiana*. The arrow indicated the non-specific amplification, "▷◁" indicated the convergent primer pairs, "◁▷" indicated the divergent primer pairs.

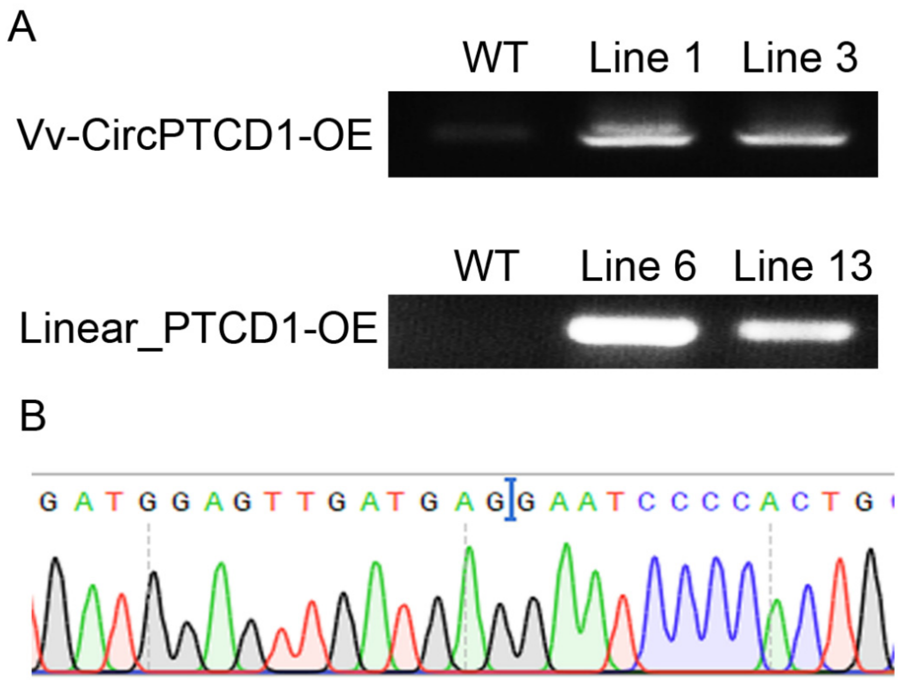

Figure S4. verification of *Vv-circPTCD1* and Linear_PTCD1 OE lines in *Arabidopsis.* (A) The gel electrophoresis of PCR amplification; (B) confirmation of back-splicing site of *Vv-circPTCD1* in ectopic OE lines.

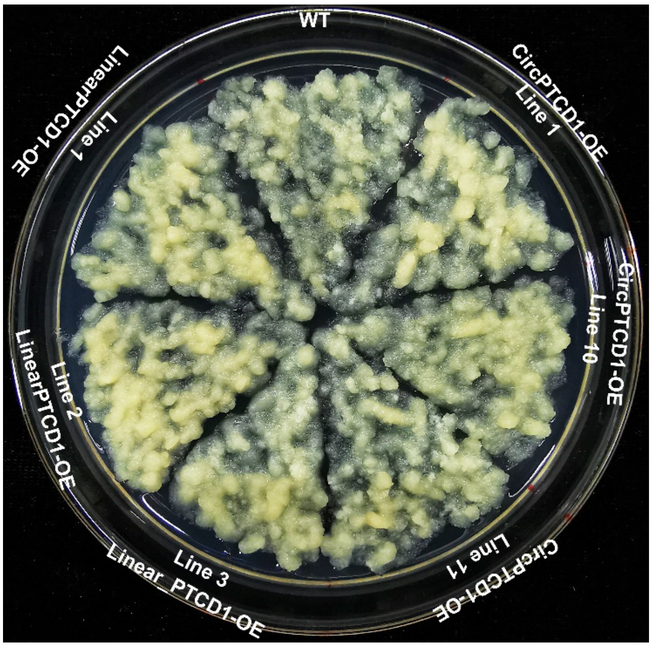

Figure S5. the phenotype of callus incubated in normal condition. The overexpressed callus mass and WT were incubated at 26°C on the dark and the phenotypic difference was undetectable.
